# Les cardiopathies de l’enfant au CHU Souro Sanou de Bobo-Dioulasso: aspects échocardiographies et thérapeutiques

**DOI:** 10.11604/pamj.2016.25.62.9508

**Published:** 2016-10-03

**Authors:** Somnoma Jean-Baptiste Tougouma, Senkaye-Lagom Aimé Kissou, Aimé Arsène Yaméogo, Nobila Valentin Yaméogo, Aimé Bama, Makoura Barro, Arsène Héma, Larissa Kaguembèga, Boubacar Nacro

**Affiliations:** 1 Institut Supérieur des Sciences de la Santé, Université Polytechnique de Bobo-Dioulasso, Burkina Fasso; 2 Service de Cardiologie, CHUSS de Bobo-Dioulasso, Burkina Fasso; 3 Service de Pédiatrie, CHUSS de Bobo-Dioulasso, Burkina Fasso; 4 Unité de Formation et de Recherche en Sciences de la Santé, Université de Ouagadougou, Burkina Fasso; 5 Centre Hospitalier Pédiatrique Charles de Gaulle de Ouagadougou, Burkina Fasso; 6 Centre Hospitalier Universitaire Souro Sanou (CHUSS), Burkina Fasso

**Keywords:** Cardiopathies congénitales, cardiopathies acquises, Bobo-Dioulasso, Congenital heart diseases, acquired heart diseases, Bobo-Dioulasso

## Abstract

Les cardiopathies de l'enfant déterminent en Afrique un problème de santé publique difficile à prendre en charge, de part la densité de la population jeune, le faible niveau socioéconomique et l'insuffisance de plateaux techniques adaptés. Les auteurs rapportent les aspects échocardiographiques et thérapeutiques des cardiopathies de l'enfant dans le département de pédiatrie du CHUSS de Bobo-Dioulasso. Il s'agissait d'une étude transversale descriptive concernant la période de janvier 2013 à décembre 2014 (24 mois). Elle a consisté en une revue documentaire des comptes rendus d'échocardiographies réalisées chez les enfants de moins de 15 ans dans le laboratoire d'échocardiographie du CHUSS et de leurs dossiers de suivi thérapeutique. Durant la période d'étude, 184 examens écho-cardiographiques avaient été réalisés et permis la mise en évidence de 93 cas de cardiopathies de l'enfant, soit 50,50% des cas. Parmi eux, on distinguait 71% (66/93) de cardiopathies congénitales et 29% (27/93) de cardiopathies acquises. Les cardiopathies congénitales les plus fréquentes étaient : la CIV (27,2%), CIA (10,6%), CAV (7,5%), T4F (9,1%), TAC (6%), formes associées (15%). Les cardiopathies acquises étaient dominées par les valvulopathies rhumatismales (48%), la cardiomyopathie dilatée hypokinétique (33,3%) et la tamponnade péricardite (18,5%). L'indication chirurgicale était posée dans 53,7% (50/93) des cas dont 86% (43/50) de cardiopathies congénitales et 14% (7/50) de cardiopathies acquises. 21% (9/43) des cardiopathies congénitales ont bénéficié d'une chirurgie cardiaque. Aucune cardiopathie acquise d'indication thérapeutique chirurgicale n'avait été opérée. Les cardiopathies de l'enfant sont fréquentes à Bobo-Dioulasso. La conception de stratégies multidisciplinaires associées à une optimisation des moyens devraient améliorer la prise en charge de ces cardiopathies.

## Abstract (en)

Children's heart diseases in Africa represent a public health problem that is difficult to cope with due to young population density, low socioeconomic status and lack of suitable technical platforms. This study aims to highlight the echocardiographic and therapeutic aspects of cardiopathies in children hospitalized at the pediatric department of the University Hospital Souro Sanou, Bobo-Dioulasso. We conducted a descriptive cross-sectional study over the period January 2013-December 2014 (24 months). It was based on a literature review of echocardiography reports performed on children under 15 years of age in the echocardiography laboratory of CHUSS and of medical records of their therapeutic follow-up. During the study period, 184 echocardiographic examinations were performed and allowed identification of 93 cases of children with heart disease (50.50% of cases). Among them, 71% (66/93) of cases with congenital cardiopathies were distinguished and 29% (27/93) of acquired cardiopathies. The most common congenital heart diseases were: IVC (27.2%), IAC (10.6%), AVC (7.5%), F4T (9.1%), CAT (6%), related forms ( 15%). Acquired heart diseases were dominated by rheumatic valve diseases (48%), hypokinetic dilated cardiomyopathy (33.3%) and pericardial tamponade (18.5%). Surgery was recommended in 53.7% (50/93) of cases, of whom 86% (43/50) with congenital heart diseases and 14% (7/50) with acquired heart diseases. 21% (9/43) of patients with congenital heart diseases underwent cardiac surgery. No acquired heart disease requiring surgery was surgically treated. Cardiopathies in children are frequent in Bobo-Dioulasso. Multidisciplinary strategies associated with resource optimization should improve the management of these cardiopathies.

## Introduction

Les cardiopathies de l'enfant déterminent en Afrique un problème de santé publique. Leur prise en charge s'avère souvent difficile du fait du faible niveau socio économique des populations et de l'insuffisance de plateaux techniques adaptés. L'échocardiographie Doppler est de nos jours l'examen indispensable pour le diagnostic, les indications thérapeutiques et la surveillance des cardiopathies de l'enfant. Au Burkina Faso plusieurs études rétrospectives ont été réalisées à Ouagadougou rapportant des prévalences variables tant en cardiologie pédiatrique que adulte [1, 2]. Dans le département de pédiatrie du CHU Souro Sanou (CHUSS), aucune étude n'a encore évalué les cardiopathies de l'enfant depuis la mise en place d'un laboratoire d'échocardiographie. Une meilleure connaissance des cardiopathies de l'enfant en milieu hospitalier pédiatrique ainsi que leur devenir thérapeutique devraient permettre d'optimiser leur prise en charge. Les auteurs proposent à travers ce travail, une description des aspects écho-cardiographiques et thérapeutiques des cardiopathies de l'enfant dans le département de pédiatrie du CHUSS de Bobo-Dioulasso.

## Méthodes

Il s'agissait d'une étude descriptive. Les données ont été recueillies à partir des comptes rendus d'échocardiographies réalisées, de janvier 2013 à décembre 2014 (24 mois), dans le laboratoire d'échocardiographie du CHUSS de Bobo-Dioulasso. Les échocardiographies ont été faites par un cardiologue échocardiographiste ayant reçu une formation en cardio-pédiatrie. Dans le laboratoire, les examens sont réalisés de façon standardisée et les comptes rendus rédigés et archivés en temps réel. Nous avons utilisés une sonde cardiaque de 5MHz sur appareil Aloka Prosound 4000 Plus pourvu de doppler pulsé, continu et couleur. Etaient inclus tous les enfants âgés de 0 à 15 ans chez qui l´échocardiographie-doppler était demandée pour suspicion de cardiopathie et dont le compte-rendu contenait des renseignements sur l´identité du patient, la date de réalisation de l´examen, l´indication de l´examen, les données de l´examen écho-cardiographique. Les données sur les aspects thérapeutiques, notamment le devenir chirurgical, ont été recueillies à partir des dossiers médicaux des patients en pédiatrie. Les données saisies sous Epidata, ont été analysées grâce au logiciel STATA^®^ 13. Les variables quantitatives ont été décrites par les médianes (me) et leurs interquartiles (IIQ) et les variables qualitatives par leur proportion (%). Nous avons utilisé le test exact de Fisher pour la comparaison des données qualitatives et le test de Student pour celle des données quantitatives. Le seuil de significativité de p était de 0,05.

## Résultats

**Fréquence globale:** Durant la période d'étude, 184 examens écho-cardiographiques ont été réalisés chez autant d'enfants, et ont permis la mise en évidence de 93 cas de cardiopathies de l'enfant. Parmi eux, on distinguait 71% (66/93) de cardiopathies congénitales et 29% (27/93) de cardiopathies acquises. Les cardiopathies de l'enfant représentaient 0,61% des 15228 admissions dans le service de pédiatrie du CHUSS pendant la période d'étude. Les cardiopathies congénitales étaient significativement plus important que les cardiopathies acquises avec respectivement 0,43% et 0,18% (p<10^-3^). La population d'étude était constituée de 97 garçons et 87 filles soit un sexe ratio de 1,11 et l'âge médian était de 42,5 mois IIQ (8,5-96). Le sexe ratio était de 1,4 pour les cardiopathies congénitales et de 1,7 pour les cardiopathies acquises. Le Tableau 1 donne la répartition des cas selon le ou les indications de l'échocardiographie. Il y ressortait que le souffle cardiaque était le motif le plus fréquent dans 32% des cas, suivi de la dyspnée d'effort 21,4% et de la cardiomégalie radiologique dans 18,5% des cas.

**Tableau 1:** Distribution des indications de l’échocardiographie de 184 enfants reçus dans le laboratoire d’échocardiographie du CHUSS de Bobo-Dioulasso (Burkina Faso), 2013-2014

Motifs de demande	Fréquence	Pourcentage (%)
Souffle cardiaque	90	32,14
Dyspnée	60	21,43
Cardiomégalie radiologique	52	18,57
Dépistage	32	11,43
Toux	19	6,79
Cyanose	10	3,57
Retard de croissance	6	2,15
Insuffisance cardiaque	4	1,43
Détresse respiratoire	3	1,07
Douleurs thoraciques	2	0,71
Syndrome polymalformatif	2	0,71
**Total**	**280**	**100**

**Les cardiopathies congénitales:** L'échocardiographie a permis de mettre en évidence 66 cas de cardiopathies congénitales avec 86 entités nosologiques. Les cardiopathies congénitales les plus fréquentes étaient : CIV (27,2%), CIA (10,6%), CAV (7,5%), T4F (9,1%), TAC (6%), formes associées (15%) (Tableau 2). La répartition nosologique des cas de cardiopathies congénitales selon le sexe montrait que les CIV étaient les plus représentées avec 27 cas (33,33%), suivies des CIA avec 8 cas (9,87%), la T4F avec 6 cas (7,4%) et le CAV complet dans 5 cas (6,17%) (Tableau 3). Les CIV de type 2b représentaient 14 cas soit 51,8% de l'ensemble des CIV. Nous avons noté un cas de CIV de type 3. L'âge médian de dépistage des cardiopathies congénitales était de 20 mois IIQ (6-60). La tranche d'âge de 01 à 30 mois était la plus représentée avec 35 cas (53,03%) suivie de celles de 30 à 60 mois et de plus de 60 mois avec respectivement 15 cas (22,72%) chacune (Tableau 4). Un seul cas de cardiopathie congénitale a été dépisté chez le nouveau-né.

**Tableau 2:** Typologie des cardiopathies congénitales diagnostiquées à l’échocardiographie au CHUSS de Bobo-Dioulasso (Burkina Faso), 2013-2014

Type de cardiopathie congénitale	Effectif	Pourcentage
CIV	18	27,2%
T4F	6	9,1%
TAC	4	6%
CAV complet	5	7,5%
CIA ostium secondum	7	10,6%
Laubry et Pezzi	3	4,5%
CIV+PCA	1	1,5%
CIV+ membrane supra pulmonaire	1	1,5%
CIV +Membrane sous aortique sténosante	1	1,5%
Laubry et Pezzi+ Membrane sous aortique sténosante	1	1,5%
Laubry et Pezzi+ Membrane sous aortique sténosante+ PCA	1	1,5%
CIV+Atrésie tricuspide+ Oreillette Unique	1	1,5%
Coarctation aorte +CMD hypokinétique	2	3%
CIA+ Sténose valvulaire pulmonaire	1	1,5%
VDDI corrigé+ Sténose sous aortique très serrée+ PCA	1	1,5%
CAV complet + PCA	1	1,5%
CAV complet+ sténose étage AP	1	1,5%
VU	1	1,5%
ASIA+FOP	2	3%
HTAP primitive	5	7,5%
Floppy valve	1	1,5%
CMH	1	1,5%
Rétrécissement sous aortique	1	1,5%
**Total**	**66**	**100%**

**Tableau 3:** Répartition des entités nosologiques en fonction du sexe au CHUSS de Bobo-Dioulasso (Burkina Faso), 2013-2014

Type de cardiopathie	Sexe		
	Masculin	Féminin	Total
CIV	18	9	27
Type 1	5	0	
Type 2a	2	5	
Type 2b	10	4	
Type 3	1	0	
T4F	3	3	6
TAC	1	3	4
CAV complet	2	3	5
Atrésie tricuspide à SIA ouvert	2	0	2
CIA ostium secondum	6	2	8
Coarctation aorte	1	1	2
IM congénitale	0	1	1
VDDI	0	1	1
TGV corrigée	1	0	0
PCA	2	2	4
RVPA partiel	1	0	1
Membrane sous aortique sténosante	1	1	2
Rétrécissement supra aortique	0	1	1
Rétrécissement sous aortique	0	1	1
Membrane supra pulmonaire sténosante	0	1	1
Ventricule Unique	0	2	2
Laubry et Pezzi	1	2	3
Sténose pulmonaire étagée	1	0	1
Sténose valvulaire pulmonaire	0	1	1
HTAP primitive	1	4	5
Oreillette Unique	1	1	2
CMH	0	1	1

**Tableau 4:** Répartition des différents types de cardiopathies congénitales en fonction la tranche d’âge au CHUSS de Bobo-Dioulasso (Burkina Faso), 2013-2014

	Tranche d’âge (mois)				
Type de cardiopathie	<1	[1-30]	30-60]	>60	Total
CIV	0	11	6	1	18
CIA	0	4	2	1	7
T4F	0	1	4	1	6
TAC	0	3	0	1	4
CAV	0	4	1	0	5
Laubry et Pezzi	0	2	0	1	3
Coarctation aorte	0	0	0	2	2
HTAP	0	0	0	5	5
ASIA+FOP	0	2	0	0	2
CIV+PCA	0	1	0	0	1
CIV+ sténose pulmonaire	0	0	0	1	1
CIV + Membrane sous aortique sténosante	0	1	0	0	1
Laubry et Pezzi+ Membrane sous aortique + PCA	0	1	0	0	1
Laubry et Pezzi+ Membrane sous aortique sténosante	0	1	0	0	1
Atrésie tricuspide +CIV + Oreillette unique	0	1	0	0	1
CIA + RP	0	0	1	0	1
VDDI corrigé+ Sténose sous aortique + PCA	0	0	0	1	1
CAV+PCA	0	1	0	0	1
CAV + sténose pulmonaire étagée	0	1	0	0	1
VU	0	1	0	0	1
CMH	1	0	0	0	1
Rétrécissement aortique	0	0	0	1	1
Floppy Valve	0	0	1	0	1

**Les cardiopathies acquises:** Le Tableau 5 nous donne la répartition des cardiopathies acquises selon le sexe. Les cardiopathies acquises étaient dominées par les valvulopathies rhumatismales dans 13 cas (48%), suivie des cardiomyopathies dilatées (CMD) hypokinétique dans 9 cas (33,3%) et les tamponnades péricardiques dans 5 cas (18,5%). L'âge médian de survenue des cardiopathies acquises était de 84 mois IIQ (48-132).

**Tableau 5:** Répartition des cardiopathies acquises selon le sexe au CHUSS de Bobo-Dioulasso (Burkina Faso), 2013-2014

Type de cardiopathie	Sexe			
	Masculin	Féminin	Effectif	Pourcentage
IM rhumatismale	4	4	8	29,6%
IA rhumatismale	1	0	1	3,7%
IT	1	0	1	3,7%
Maladie Mitrale	2	0	2	7,4%
RM acquis	0	1	1	3,7%
CMD hypokinétique	5	4	9	33,3%
Tamponnade péricardique	4	1	5	18,5%
**Total**	**17**	**10**	**27**	**100%**

**Devenir chirurgical:** L'indication chirurgicale était posée dans 53,7% (50/93) dont 86% (43/50) des cas de cardiopathies congénitales et 14% (7/50) des cas de cardiopathies acquises. Vingt et un pour cent (9/43) des cardiopathies congénitales ont bénéficié d'une chirurgie cardiaque. Aucune cardiopathie acquise d'indication thérapeutique chirurgicale n'a été opérée.

## Discussion

### Limites de l'étude

Elles sont inhérentes au caractère rétrospectif de notre étude. Seuls les cas ayant bénéficié d'une échocardiographie au CHUSS ont été inclus dans le présent travail, ce qui a entraîne probablement une sous-estimation de la fréquence de cardiopathies de l'enfant. De plus, cette étude menée uniquement en milieu hospitalier ne peut refléter l'ampleur réelle des cardiopathies de l'enfant dans la communauté. Malgré ces limites, les résultats auxquels nous sommes parvenus nous ont inspiré quelques commentaires et nous avons pu également les comparer à ceux d'autres auteurs.

### Les cardiopathies congénitales

Dans notre étude, les cardiopathies congénitales représentaient 71% des cardiopathies de l'enfant et 0,61% de l'ensemble des admissions dans le département de pédiatrie du CHUSS. Bien que supérieur au 0,1% rapportée par Amon-Tanoh-Dick en Côte d'Ivoire dans une population de nouveau-nés [3], notre fréquence reste dans la fourchette de données rapportées par les différentes séries africaines en milieu pédiatrique qui varient de 0,48% à 0,98% [1, 4–6] en milieu pédiatrique. Le sexe masculin prédominait dans notre travail avec un sex-ratio de 1,4. Cette proportion était comparable aux données de la plupart des séries africaines qui rapportent toutes une prédominance du sexe masculin [4, 7, 8]. L'âge médian des enfants porteurs de cardiopathies congénitales dans notre série était de 20 mois IIQ (6-60) et la tranche d'âge la plus représentée était celle de 1-30 mois avec 35 cas (53,03%). Notre proportion est comparable à celles rapportées par Kinda à Ouagadougou [1], Cloarec en France [9] et Abéna [6] à qui retrouvaient respectivement 55, 61 et 70% des cardiopathies congénitales dans cette tranche d'âge. Cela pourrait s'expliquer par la prédominance des cardiopathies congénitales de type shunt gauche droite (69,7% dans notre série). En effet, le retard de maturation pulmonaire, phénomène classique à la naissance retarde l'apparition des premiers signes, qui surviennent généralement après un mois de vie, les enfants devenant symptomatique amenant les parents à la consultation et au diagnostic de ses cardiopathies. Douze cas (18,18%) de cardiopathies congénitales ont été découverts après 60 mois, dont 4 cas de CIV. Cela pourrait s'expliquer par le retard à la consultation et à l'errance diagnostique dans un contexte de difficultés d'accès aux soins et au manque de personnels compétents. Ces retards diagnostics sont sources de mauvais pronostic, en témoigne le cas de CIV de type 3 retrouvée dans notre série. La CIV est la cardiopathie congénitale la plus fréquente dans notre série avec 27,2% des cas. Cette proportion est comparable à celles retrouvées dans la plupart des séries africaines [3, 4, 6–8, 10–13] et dans le monde [14, 15]. Elle était à prédominance péri membraneuse et le type 2b était prépondérant avec 14 cas (soit 51,85%). Cette forme typologique est celle d'indication chirurgicale par excellence, car une évolution vers la fermeture spontanée est rare alors que le risque d'évolution rapide vers une forme de type 3 de mauvais pronostic est à redouter [16].

### Les cardiopathies acquises

Dans notre série, les cardiopathies acquises représentaient 29% de l'ensemble des cardiopathies de l'enfant et 0,18% de l'ensemble des admissions dans le département de pédiatrie. Elles étaient significativement moins fréquentes que les cardiopathies congénitales (0,61% Vs 0,18% ; p<10^-3^). Ce constat est rapporté par plusieurs auteurs africains depuis quelques années [17–19]. Pendant longtemps considérée comme la pathologie prédominante dans les pays en développement, on assiste de nos jours à un recul des cardiopathies acquises de l'enfant notamment les valvulopathies post rhumatismale (48% dans notre série). L'explication serait multifactorielle. D'une part, l'amélioration des conditions de vie des populations diminue les cas d'infections streptococciques à l'origine des cardiopathies rhumatismales. D'autre part, la contribution des politiques de santé dans les pays en développement comme l'Initiative de Bamako permettant des soins de santé primaires de qualité, et notamment une accessibilité aux antibiotiques.

### Devenir chirurgical

La prise en charge chirurgicale reste insuffisante dans notre série avec 21% de corrections chirurgicales pour les cardiopathies congénitales et aucun cas de chirurgie des cardiopathies acquises. Ces chiffres témoignent du caractère préoccupant des cardiopathies de l'enfant en Afrique. La chirurgie cardiaque est pour l'instant inexistante au Burkina Faso. Les évacuations sanitaires sont assurées en majorité par les associations mécènes. Ce constat est rapporté dans la plupart des pays d'Afrique Subsaharienne [19–21]. Dans notre série, l'ensemble des enfants opérés ont bénéficié de cette collaboration. Il serait judicieux pour les pays d'Afrique subsaharienne de fédérer les moyens afin d'offrir un plateau technique chirurgical sous régional pour une prise en charge des cardiopathies de l'enfant dont les coûts seraient acceptables pour l'ensemble des pays.

## Conclusion

Les cardiopathies de l'enfant sont fréquentes à Bobo-Dioulasso. Elles sont dominées par les cardiopathies congénitales. Leur prise en charge chirurgicale reste insuffisante. La conception de stratégies multidisciplinaires associées à une optimisation des moyens des pays d'Afrique Subsaharienne pourraient améliorer la prise en charge de ces cardiopathies.

### Etat des connaissances actuelles sur le sujet

- Le dépistage des cardiopathies est bien codifié dans les pays développés avec un volet anténatal bien développé grâce aux nouvelles techniques d'ultrasonographie;
- La prise en charge par correction chirurgicale est la règle dans les pays développés. Cette chirurgie cardiaque est encore au stade embryonnaire dans nos pays.

### Contribution de notre étude à la connaissance

- La prévalence des cardiopathies de l'enfant est connue à Bobo-Dioulasso;
- La prédominance des cardiopathies congénitale est établie et peu d'enfants porteurs de cardiopathies opérables bénéficient de cette chirurgie;
- Amélioration des politiques sanitaires et une optimisation des moyens des pays Africains pour la mise en place d'un plateau technique adéquat.
